# Lower endothelium-dependent microvascular function in adult breast cancer patients receiving radiation therapy

**DOI:** 10.1186/s40959-021-00104-z

**Published:** 2021-05-13

**Authors:** Heather R. Banister, Stephen T. Hammond, Shannon K. Parr, Shelbi L. Sutterfield, Vanessa-Rose G. Turpin, Scott Treinen, Martin J. Bell, Carl J. Ade

**Affiliations:** 1 Clinical Integrative Physiology Laboratory, Department of Kinesiology, College of Health and Human Sciences, Kansas State University, Manhattan, KS 66506 USA; 2 Central Kansas Cancer Center, Manhattan, KS USA; 3 Johnson Cancer Research Center, Kansas State University, Manhattan, KS 66506 USA

**Keywords:** Endothelium, Vasodilation/radiation effects

## Abstract

**Purpose:**

Cancer patients with a history of radiotherapy are at an increased risk of ischemic heart disease. Preclinical animal studies demonstrate markedly impaired acetylcholine (ACh)-mediated endothelium-dependent vasorelaxation within days to weeks post-irradiation, however, whether microvascular function is affected in the intact human circulation during cancer radiation therapy has yet to be determined.

**Materials and methods:**

Using laser-Doppler flowmetry, microvascular endothelium-dependent and independent responses were evaluated through iontophoresis of acetylcholine (ACh) (*part 1*, *n* = 7) and sodium nitroprusside (SNP) (*part 2*, *n* = 8), respectively, in women currently receiving unilateral chest adjuvant radiation therapy for breast cancer. Measurements were performed at the site of radiation treatment and at a contralateral control, non-radiated site. Cutaneous vascular conductance (CVC) was calculated by normalizing for mean arterial pressure.

**Results and Conculsions:**

In *part 1*, patients received an average radiation dose of 2104 ± 236 cGy. A significantly lower peak ACh-mediated endothelium-dependent vasodilation was observed within the radiated microvasculature when compared to non-radiated (radiated: 532 ± 167%, non-radiated 1029 ± 263%; *P* = 0.02). In *part 2*, the average radiation dose received was 2251 ± 196 cGy. Iontophoresis of SNP elicited a similar peak endothelium-independent vasodilator response in radiated and non-radiated tissue (radiated: 179 ± 58%, non-radiated: 310 ± 158; *P* = 0.2). The time to 50% of the peak response for ACh and SNP was similar between radiated and non-radiated microvasculature (*P* < 0.05). These data provide evidence of early endothelium-dependent microvascular dysfunction in cancer patients currently receiving chest radiation and provide the scientific premise for future work evaluating coronary endothelial function and vasomotor reactivity using more detailed and invasive procedures.

## Introduction

Long-term follow-up studies in breast cancer survivors demonstrate a significant increased risk of ischemic heart disease following radiotherapy, with reports suggesting a 7.4% increased risk of ischemic heart disease per Gy within 5 years post-exposure and that extends beyond 20 years, independent of other risk factors [1]. This increased risk of cardiovascular complications, presumably through incidental irradiation of the heart and surrounding vasculature, highlights the importance of understanding the pathophysiology or radiation-induced cardiotoxicity.

Exposure of animal subjects in pre-clinical studies to a range of radiation doses markedly impairs acetylcholine (ACh)-mediated endothelium-dependent vasorelaxation within a days-to-weeks post-irradiation [2–4]. Specifically, Menendez et al. [2] observed a significant impaired relaxation response to ACh in thoracic artery segments in rats exposed to less than 0.2 cGy within 3 days. This response, despite the very low radiation, demonstrates that adverse vascular consequences may be occurring early in a cancer patient’s treatment. In support of this, fractionated radiation (250 cGy dose) in rats resulted in decreased endothelium-dependent dilation observed after only a second radiation dose. This work in preclinical models support the premise that radiation elicits vascular endothelium injury at even low radiation dosages. However, a limited number of studies in human patients are available to confirm these observations. In breast cancer survivors with a history of unilateral radiation therapy, Beckman et al. [5] observed a decreased flow-mediated endothelium-dependent dilation in radiated axillary arteries compared with the contralateral, non-radiated arteries. Moreover, they observed an increased endothelium-independent vasodilation, further supporting an impaired endothelial function following radiotherapy. These findings compliment work from Sugihara et al. [6] that demonstrated, in vitro, a decreased endothelium-dependent vasodilation, but unchanged endothelium-independent dilation, in cervical arteries taken from cancer survivors exposed to radiation. These initial studies in axillary and cervical arteries provide valuable insight into endothelial dysfunction elicited by radiation therapy in cancer survivors years after exposure. However, there is a paucity in our understanding regarding the pathophysiology of radiation-induced vascular dysfunction in the early (i.e., within days of exposure) radiation treatment process, particularly within the microcirculation. This is a critical knowledge gap given decreases in microvascular function occur early in the progression of numerous cardiovascular and metabolic diseases [7–9]. As such, the evaluation of microvascular function in patients actively receiving radiation therapy will provide valuable insight into the adverse cardiovascular adaptations that occur with radiation exposure. The primary aim of this study was, therefore, to determine the acute effects of localized radiation treatment on cutaneous microvascular function in breast cancer patients. We hypothesized that the endothelium-dependent response to acetylcholine (ACh), but not endothelium-independent responses to sodium nitroprusside (SNP), would be blunted within radiated tissue when compared to the contralateral, non-radiated tissue in breast cancer patients. We chose to evaluate cutaneous microvascular function as it has previously been used to better understand the pathophysiological role of vascular dysfunction in heart failure, atherosclerosis, coronary artery diseases, peripheral vascular diseases, type 2 diabetes, and chemotherapy-induced cardiotoxicity [10–15]. In addition, the skin receives a high dose of radiation during treatment and, therefore, is more susceptible to radiation-induced injury [16].

## Methods

### Participants

All procedures were approved by the Institutional Review Board of Kansas State University and conformed to the standards set by the Declaration of Helsinki. Written informed consent was obtained from all patients prior to participation in this study. Fifteen postmenopausal women currently receiving unilateral chest adjuvant radiation therapy for breast cancer participated in the study. All patients received three-dimensional conformal radiation therapy using a tangential photon irradiation procedure. In all cases the treatment was delivered in daily fractionated doses (200–250 cGy). All patients were free of known atherosclerotic cardiovascular disease and diabetes determined by medical history. Cancer diagnosis, site of radiation treatment, cumulative dosage, duration, and use of adjuvant chemotherapy were confirmed by each patient’s treating oncologist and radiologist.

### Measurement protocol

All measurements were performed following at completion of ≥5 days of radiation treatment and ≥ 2 h following their most recent treatment session. Allowing time between treatment and measurements is essential as Tesselaar et al. [17] demonstrated a significant increase in basal skin blood flow when measured immediately following radiation treatment lateral to the areola of the irradiated breast. This previous work highlights the acute hyperemic response to radiation alone following exposure. To determine if a hyperemic response exist beyond 2 h of treatment, we compared baseline resting cutaneous blood flows between irradiated and non-radiated tissues. For the duration of the measurements the patient was placed in an outpatient setting in a quiet, temperature controlled room (21–23 °C) with measurements performed after 20 min of acclimation in the supine position. The infraclavicular regions of both the radiated and contralateral non-radiated sides of the chest were exposed and cleaned with 70% Isopropyl Alcohol. An iontophoresis drug delivery probe with integrated laser Doppler flowmeter (PeriFlux 5010 laser-Doppler perfusion monitor; Perimed, Jarfalla, Sweden) and temperature regulator were placed on the lower portion of the infraclavicular region over the 3rd or 4th intercostal space along the mid-clavicular line, approximately 15 cm away from a conductive hydrogel drug-dispersive electrode (PF 384; Perimed, Järfälla, Sweden). The laser Doppler flowmeter allowed for continuous measurements of cutaneous red blood cell flux, which was used as an index of cutaneous blood flow (CBF) and was calibrated according to the manufacturers’ specification with Brownian motility standard solution. The temperature regulator located around the perimeter of the probe maintained local skin temperature of 33 °C throughout the duration of the test. Intensity, duration, and intervals of the current delivery were controlled by a USB power supply (PF 751, Perimed, Järfälla, Sweden) connected to both the drug dispersive and drug delivery probes and were was managed and confirmed with the systems software (PeriIont Software; Perimed). Throughout the test, beat-by-beat blood pressure was continuously measured throughout during each visit via photoplethysmography (Finometer Pro, Finapres Medical Systems, Amsterdam, The Netherlands). Unless restricted by lymph node dissection or lymphedema, blood pressure was performed on the right side at heart level.

### Iontophoresis drug delivery

To assess endothelium-dependent dilation (*part 1*), 200 μL of a 2% ACh solution was applied to an iontophoresis drug delivery probe. The ACh protocol consisted of a baseline measurement followed by seven current pulses (100-μA anodal current) of 20 s, separated by 60 s current-free intervals. This delivery protocol is consistent with our previous work and has been shown to elicit no detectable current-induced axon-mediated vasodilation [14, 18]. To determine endothelium-independent dilation (*part 2*), 200 μL of a 1% SNP solution was administered. The SNP protocol consisted of a baseline measurement followed by eight current pulses (20-μA cathodal current) of 60 s, separated by 2 min current-free intervals. This protocol has been shown to limit the extent of non-specific vasodilation when used with SNP [19].

For *part 1* and *part 2* CBF was recorded at 100 Hz by data acquisition software (DI-720, DATAQ Instruments, Akron, OH, USA) and reported in arbitrary perfusion units (PU). To normalize for mean arterial pressure (MAP), cutaneous vascular conductance (CVC, PU/mmHg) was calculated as: (PU/MAP) × 100. The CBF and CVC responses averaged over 1 min at baseline and every 10 s during the charge protocol and 5 min into recovery to ensure the peak vasodilatory response was reached. The results for both *part 1* and *part 2* are presented as the relative change in CVC from baseline to each peak dose response, calculated as: [(peak-baseline CVC)/baseline CVC] × 100, and the time to 50% of the peak response (TTP50).

### Statistical analysis

Statistical analyses were performed using a commercially available software package (Prism 8, GraphPad Software, San Diego, CA, USA). The study was a priori powered based on a given Cohen’s d (i.e. effect size (ES)) to obtain sufficient power to detect differences in the peak ACh response. Based on our previous work, an effect size of 0.8 [14], with a 5% level of significance, the total required sample size for reaching the minimum power of 0.8 was 7–8 patients. The peak ACh and SNP responses were determined by paired t-tests. The entire responses to ACh and SNP iontophoresis were compared between patients with 2-way repeated measures ANOVA. To control for variability in resting baseline CBF, ANCOVA analysis was also used to detect difference in the peak CBF response with baseline CBF as a confounding variable. To minimize the chances of a type II error due to a modest sample size, ESs were calculated for each primary outcome as Cohen’s d, which provides information on the magnitude of the difference between the groups. The threshold values for ES were defined as small, moderate, and large effects as 0.2, 0.5, and > 0.8, respectively [20]. All data are presented as mean ± SE, unless otherwise stated. Statistical significance was declared at *P* < 0.05.

## Results

### Part 1

Seven patients, aged 66 ± 2 years, undergoing radiation therapy were enrolled. Patients had a resting MAP of 99 ± 3 mmHg, BMI of 28 ± 2 kg/m^2^, and had received 10 ± 1 fractionated doses of therapy treatment. The average cumulative dose of radiation received was 2104 ± 236 cGy. Three patients had received chemotherapy prior to the initiation of radiation treatment. Three patients were on either statins or hypertension medication at the time of the study.

**Fig. 1:**
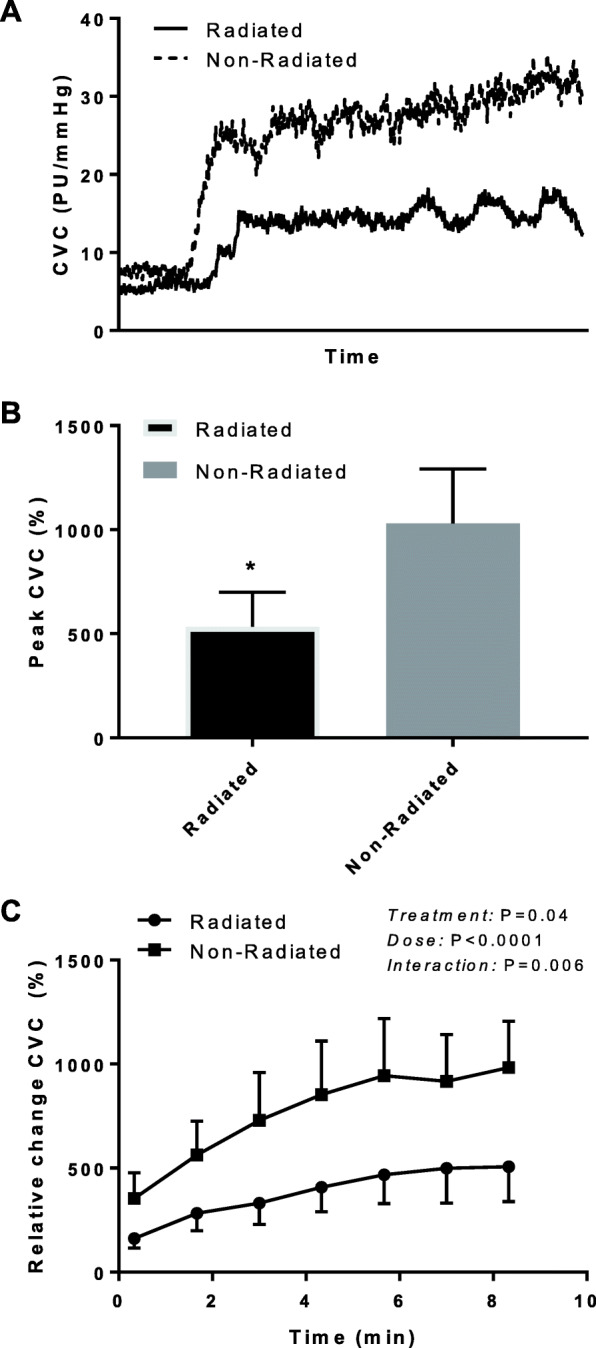
**a** Cutaneous vascular conductance (CVC) response to ACh iontophoresis in radiated and non-radiated tissue in a representative cancer patient. **b** Peak CVC response to ACh iontophoresis in radiated and non-radiated tissue in a representative cancer patient. **c** Relative increase in cutaneous vascular conductance (%CVC) in response to incremental ACh iontophoresis in cancer patients currently receiving radiation therapy. %CVC was significantly lower in the radiated tissue compared to non-radiated tissue. * Denotes significantly different vs. non-radiated. Values are mean ± SE; *n* = 7

A representative trace of the ACh cutaneous microvascular function protocol is illustrated in Fig. 1a. Baseline CVC in the cancer patients was 11.0 ± 4.1 PU/mmHg and 10.7 ± 2.9 PU/mmHg for radiated and non-radiated tissue, respectively (*P* = 0.96). Similarly, baseline CBF was not different between radiated and non-radiated tissue (*P* = 0.4). The peak vasodilatory response to ACh was significantly lower in the radiated microvasculature compared to non-radiated (Fig. 1b) (radiated: 532 ± 167%, non-radiated 1029 ± 263%; *P* = 0.02, ES = 0.7). Similarly, ANCOVA with baseline CBF as a covariate revealed a lower peak absolute CBF response to ACh in the radiated microvasculature compared to non-radiated (*P* = 0.04), but with a lower effect size (ES = 0.3). A significantly lower endothelium-dependent vasodilation was observed with ACh administration across the whole iontophoresis protocol within the radiated tissue when compared to non-radiated contralateral tissue in breast cancer patients currently receiving radiation therapy (Fig. 1c). However, the TTP50 for ACh was similar between tissues (radiated: 164.6 ± 43.8 s, non-radiated 147.7 ± 33.7 s; *P* = 0.36).

### Part 2

Eight patients, aged 56.9 ± 2.2 years, undergoing radiation therapy were studied to determine SNP-mediated endothelium-independent vascular function. These patients had a resting MAP of 97 ± 4 and had received 8.6 ± 0.7 doses of therapy treatment. The average cumulative dose of radiation received was 2251 ± 196. Two patients had received chemotherapy prior to the initiation of radiation treatment. Two patients were on statins or hypertension medication at the time of the study.

**Fig. 2:**
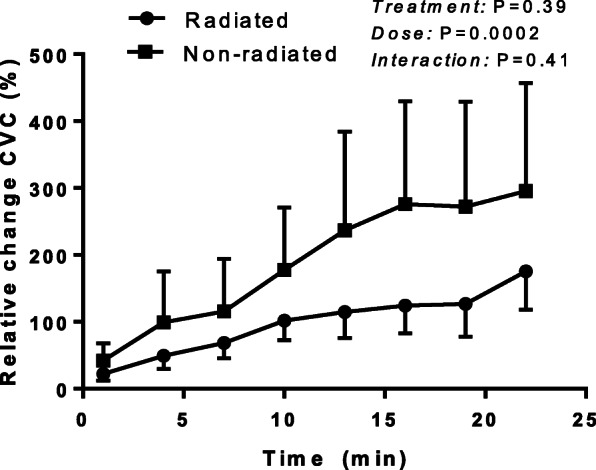
Relative increase in cutaneous vascular conductance (%CVC) in response to incremental SNP iontophoresis in cancer patients currently receiving radiation therapy. Values are mean ± SE; *n* = 8

In the patients who completed the SNP protocol, baseline CVC was 14.3 ± 3.5 PU/mmHg and 7.7 ± 1.1 PU/mmHg for radiated and non-radiated tissue, respectively (*P* = 0.12), with no difference in baseline CBF (*P* = 0.11). Peak response to iontophoresis of SNP was similar between tissues (radiated: 179 ± 58%, non-radiated: 310 ± 158; *P* = 0.2, ES = 0.3). Similarly, ANCOVA with baseline CBF as a covariate revealed a similar peak absolute CBF response to SNP in the radiated microvasculature compared to non-radiated (*P* = 0.9, ES < 1). Endothelium-independent vasodilation was similar between radiated and the contralateral non-radiated tissue across the whole iontophoresis protocol (Fig. 2). In addition, the TTP50 for SNP was similar between tissues (radiated: 556.5 ± 65.1 s, non-radiated 403.9 ± 91.0 s; *P* = 0.17).

## Discussion

The major finding in the present study was that fractionated radiation treatment in breast cancer patients, cutaneous microvascular endothelium-dependent vasoreactivity, but not endothelium-independent, was lower in radiated tissue when compared to contralateral, non-radiated tissue. These exploratory findings support the hypothesis that therapeutic radiation has an acute negative impact on microvascular endothelium-dependent vasoreactivity, which may contribute to an increased risk of adverse cardiovascular events reported following radiation therapy in cancer patients [1, 21, 22]. Given the established early role for microvascular dysfunction in the progression of numerous cardiovascular and metabolic disease [7, 8, 15], these findings provided additional insight into the changes in cardiovascular health associated with radiation therapy. While the regulation of the cutaneous circulation is somewhat different compared to other vascular tissue, previous work has proposed it as a model of generalized microvascular function [23] and has been used to predict cardiac events in clinical populations [12]. Moreover, Khan et al. [24] have demonstrated a significant correlation between Ach-mediated cutaneous microvascular reactivity and coronary vascular function. This is significant given detriments in coronary vascular function have been shown to be associated with diastolic dysfunction and heart failure [25]. The findings of the present study, therefore, are clinically significant given reports of accelerated atherosclerosis, leading to severe coronary artery disease, following chest radiotherapy [26–28]. Moreover, the findings, in a relatively modest sample size, provide the scientific premise for future work evaluating coronary endothelial function and vasomotor reactivity using more detailed and invasive procedures, such as myocardial contrast echocardiography [29].

Mechanistically, radiation exposure is hypothesized to decrease endothelial function via increased superoxide (O_2_^−^) production resulting in an attenuation in eNOS signaling by O_2_^−^ binding to NO, creating peroxynitrate (ONOO^−^) and decreasing NO bioavailability [30]. A decrease in eNOS expression has been reported in human cervical arteries radiated with an average of 4790 cGy when compared to non-radiated control vessels [6]. Similarly, others have observed a significantly decreased, but not eliminated, expression of eNOS in rabbit ear arteries following 4500 cGy irradiation when compared to non-radiated arteries at both 1 and 4 weeks post radiation (~ 69% and ~ 70% decreased relative to control, respectively) [4]. Complimenting these findings, Holler et al. [31] observed a decrease in eNOS expression following both 1000 cGy and 4500 cGy doses of radiation in mice. These findings support significant decreases in eNOS at both low and high therapeutic radiation doses, which may contribute to the decreased ACh-mediated vasodilation observed in the present study. In the present study, endothelium-dependent vasodilation to ACh was lower in the radiated cutaneous circulation compared to non-radiated tissue. Importantly, several investigations [32–35], but not all [23], have demonstrated a key role for NO in ACh-mediated vasodilation in the cutaneous circulation. While the eNOS-dependent pathway is a key, several studies have also shown that COX-dependent pathways and endothelium-derived hyperpolarizing factor (EDHF)-dependent pathways are also thought to play a role in ACh-mediated cutaneous vasodilation [23, 33–35]. As such, the findings of the present study should not be interpreted as a strict impairment in NO-mediated dilation, but simply as a degree of endothelial dysfunction within the microcirculation.

Previous research in animals exposed too high and low radiation levels have demonstrated significant decreases in endothelium-dependent vascular function [2–4, 36–38]. Hatoum et al. [36] removed submucosal intestinal arteries from radiated rats, and demonstrated an attenuated response to ACh-mediated vasodilation following the second fractional dose of 250 cGy. Importantly, they also observed high levels of superoxide within the radiated vessels when compared to those of the controls. These increased superoxide levels were observed at each radiation dose. In agreement with these findings, On et al. [3] found ACh-induced relaxation of the thoracic aorta was significantly decreased in radiated rats (1000 cGy) when compared to non-radiated controls, but was prevented when rats were supplemented with the antioxidant vitamin C [3]. This prevention of radiation induced endothelial dysfunction by pretreatment with vitamin C supports the role of oxidative damage as a potential underlying mechanism.

Currently, there is limited information on the effects of therapeutic levels of radiation on endothelium dependent vascular health in the human population. Sugihara et al. [6] examined ACh-mediated relaxation in cervical arteries taken from the neck region of cancer patients who received, on average, 4790 cGy of radiation therapy. In their study the maximum ACh-mediated vasodilatory response was significantly diminished (~ 70%) in radiated arteries compared with to controls. To our knowledge, only Beckman et al. [5] have investigated the effects of radiation therapy on the axillary artery in human breast cancer survivors, doing so, on average, 12 ± 6 years post radiation treatment. In agreement with our findings, they observed a lower endothelium-dependent flow-mediated dilation response in radiated axillary arteries when compared to contralateral non-radiated arteries. Similar to the work of Beckman et.al [5]., we compared radiated and non-radiated vasculature within the same patient, to minimize the effects of variable confounders between patients, during the radiation treatment period. Our findings of an attenuated ACh-mediated endothelium-dependent vasodilation within the radiated cutaneous microvasculature, in combination with the previous findings of Beckman et al. [5] and Sugihara et al. [6], demonstrate that radiation induces significant decreases in endothelium-dependent micro- and macrovascular function, with our findings demonstrating adverse alterations very early in the radiation treatment process. Beckman and colleagues also examined changes in endothelium-independent vasoreactivity in breast cancer patients treated with irradiation. In contrast to our findings, they reported a greater vasodilation response of irradiated auxiliary arteries after treatment with nitroglycerin when compared to their own non-radiated contralateral auxiliary arteries. The results of this study represent the long-term effects of radiation since their subjects were on average 12 years post radiation. Coupled with our acute findings, this suggests there is a time dependent response to changes in endothelium-independent function after radiation therapy.

### Experimental considerations

There are several experimental considerations regarding this study. First, some of the patients in the present study also received chemotherapy, which by itself has previously been shown to decrease cutaneous microvascular function [14]. Since the effects of chemotherapy are systemic and patients served as their own control, this allowed these patients to be included in the study. However, a key study consideration is our lack of a healthy non-cancer control group similar to our previous work and that of Beckman et al. [5]. Given the use of medications that can impact skin blood flow responses, including anti-cancer chemotherapy, by our group of patients, the ability to evaluate the effect of radiation alone, while taking into consideration external factors (e.g., stress, medication) was best achieved by evaluating irradiated tissue and non-radiated tissue in the same patient at the same time. Second, the population measured in this study was strictly female breast cancer patients, therefore these results may not be representative of cancer patient population in its entirety. However, it is worth noting that radiation treatment is one of the most commonly used therapeutic intervention for men diagnosed with prostate cancer and therefore, these patients may experience similar decreases in cutaneous microvascular function [39, 40]. Third, iontophoresis of ACh, can elicit a nonspecific axon reflex vasodilation. However, we utilized previously reported low current density delivery protocols, which eliminate the impact of nonspecific axon reflexes [14, 41–43]. We were also unable to heat the skin to evaluate cutaneous microvascular vasodilation, which is primarily NO dependent [34], due to the increased risk of injury associated with heating the radiated skin.

## Conclusions

The main conclusions of the present study is that endothelium-dependent vasoreactivity, but not endothelium-independent vasoreactivity, within cutaneous microvascular beds is attenuated in breast cancer patients currently receiving radiation therapy. These findings suggest that patients undergoing radiation therapy experience decreased endothelium-dependent vasodilator function within radiated tissue despite relatively low radiation exposure, which may contribute to the increased long-term risk of cardiovascular disease morbidity and mortality experienced by breast cancer survivors. Given the slight differences in mechanisms mediating vascular regulation in various vascular beds, future work need to focus on further validation of our findings from the skin with more direct measures of myocardial perfusion (e.g., myocardial contrast echocardiography).

## Data Availability

The datasets during and/or analyzed during the current study available from the corresponding author on reasonable request.

## Abbreviations

ACh: Acetylcholine
BMI: Body mass index
cGy: Centigray
CBF: Cutaneous blood flow
CVC: Cutaneous vascular conductance
EDHF: Endothelium-derived hyperpolarizing factor
MAP: Mean arterial pressure
NO: Nitric Oxide
PU: Perfusion units
ONOO-: Peroxynitrate
SNP: Sodium nitroprusside
O2-: Superoxide
TTP50: Time to 50% of the peak response

## References

[1] Darby SC, Ewertz M, McGale P, Bennet AM, Blom-Goldman U, Brønnum D, Correa C, Cutter D, Gagliardi G, Gigante B, Jensen MB, Nisbet A, Peto R, Rahimi K, Taylor C, Hall P (2013). Risk of ischemic heart disease in women after radiotherapy for breast cancer. N Engl J Med.

[2] Menendez JC, Casanova D, Amado JA, Salas E, García-Unzueta MT, Fernandez F, de la Lastra LP, Berrazueta JR (1998). Effects of radiation on endothelial function. Int J Radiat Oncol Biol Phys.

[3] On YK, Kim HS, Kim SY, Chae IH, Oh BH, Lee MM, Park YB, Choi YS, Chung MH (2001). Vitamin C prevents radiation-induced endothelium-dependent vasomotor dysfunction and de-endothelialization by inhibiting oxidative damage in the rat. Clin Exp Pharmacol Physiol.

[4] Qi F, Sugihara T, Hattori Y, Yamamoto Y, Kanno M, Abe K (1998). Functional and morphological damage of endothelium in rabbit ear artery following irradiation with cobalt60. Br J Pharmacol.

[5] Beckman JA, Thakore A, Kalinowski BH, Harris JR, Creager MA (2001). Radiation therapy impairs endothelium-dependent vasodilation in humans. J Am Coll Cardiol.

[6] Sugihara T, Hattori Y, Yamamoto Y, Qi F, Ichikawa R, Sato A, Liu MY, Abe K, Kanno M (1999). Preferential impairment of nitric oxide-mediated endothelium-dependent relaxation in human cervical arteries after irradiation. Circulation.

[7] Fernlund E, Schlegel TT, Platonov PG, Carlson J, Carlsson M, Liuba P (2015). Peripheral microvascular function is altered in young individuals at risk for hypertrophic cardiomyopathy and correlates with myocardial diastolic function. Am J Phys Heart Circ Phys.

[8] Patik JC, Christmas KM, Hurr C, Brothers RM (2016). Impaired endothelium independent vasodilation in the cutaneous microvasculature of young obese adults. Microvasc Res.

[9] Osborne MT, Bajaj NS, Taqueti VR, Gupta A, Bravo PE, Hainer J, Bibbo CF, Dorbala S, Blankstein R, Di Carli MF (2017). Coronary microvascular dysfunction identifies patients at high risk of adverse events across cardiometabolic diseases. J Am Coll Cardiol.

[10] Cui J, Arbab-Zadeh A, Prasad A, Durand S, Levine BD, Crandall CG (2005). Effects of heat stress on thermoregulatory responses in congestive heart failure patients. Circulation.

[11] Farkas K, Kolossváry E, Járai Z, Nemcsik J, Farsang C (2004). Non-invasive assessment of microvascular endothelial function by laser Doppler flowmetry in patients with essential hypertension. Atherosclerosis.

[12] Heitzer T, Schlinzig T, Krohn K, Meinertz T, Münzel T (2001). Endothelial dysfunction, oxidative stress, and risk of cardiovascular events in patients with coronary artery disease. Circulation.

[13] Rossi M, Carpi A (2004). Skin microcirculation in peripheral arterial obliterative disease. Biomed Pharmacother.

[14] Sutterfield SL, Caldwell JT, Post HK, Lovoy GM, Banister HR, Ade CJ (2018). J Appl Physiol (Bethesda, Md : 1985).

[15] Verma S, Buchanan MR, Anderson TJ (2003). Endothelial function testing as a biomarker of vascular disease. Circulation.

[16] Jaschke W, Schmuth M, Trianni A, Bartal G (2017). Radiation-induced skin injuries to patients: what the interventional radiologist needs to know. Cardiovasc Intervent Radiol.

[17] Tesselaar E, Flejmer AM, Farnebo S, Dasu A (2017). Changes in skin microcirculation during radiation therapy for breast cancer. Acta Oncol.

[18] Loader J, Roustit M, Taylor F, MacIsaac RJ, Stewart S, Lorenzen C, Walther G (2017). Assessing cutaneous microvascular function with iontophoresis: avoiding non-specific vasodilation. Microvasc Res.

[19] Droog EJ, Henricson J, Nilsson GE, Sjoberg F (2004). A protocol for iontophoresis of acetylcholine and sodium nitroprusside that minimises nonspecific vasodilatory effects. Microvasc Res.

[20] Vincent WJ, Weir JP (2012). Statistics in kinesiology.

[21] Hooning MJ, Botma A, Aleman BM, Baaijens MH, Bartelink H, Klijn JG, Taylor CW, van Leeuwen FE (2007). Long-term risk of cardiovascular disease in 10-year survivors of breast cancer. J Natl Cancer Inst.

[22] Seddon B, Cook A, Gothard L, Salmon E, Latus K, Underwood SR, Yarnold J (2002). Detection of defects in myocardial perfusion imaging in patients with early breast cancer treated with radiotherapy. Radiother Oncol.

[23] Holowatz LA, Thompson CS, Minson CT, Kenney WL (2005). Mechanisms of acetylcholine-mediated vasodilatation in young and aged human skin. J Physiol.

[24] Khan F, Patterson D, Belch JJ, Hirata K, Lang CC (2008). Relationship between peripheral and coronary function using laser Doppler imaging and transthoracic echocardiography. Clin Sci (London, England : 1979).

[25] Taqueti VR, Solomon SD, Shah AM, Desai AS, Groarke JD, Osborne MT, Hainer J, Bibbo CF, Dorbala S, Blankstein R, di Carli MF (2018). Coronary microvascular dysfunction and future risk of heart failure with preserved ejection fraction. Eur Heart J.

[26] Cheng YJ, Nie XY, Ji CC, Lin XX, Liu LJ, Chen XM, Yao H, Wu SH (2017). Long-term cardiovascular risk after radiotherapy in women with breast cancer. J Am Heart Assoc.

[27] Hull MC, Morris CG, Pepine CJ, Mendenhall NP (2003). Valvular dysfunction and carotid, subclavian, and coronary artery disease in survivors of hodgkin lymphoma treated with radiation therapy. Jama.

[28] Lee MS, Finch W, Mahmud E (2013). Cardiovascular complications of radiotherapy. Am J Cardiol.

[29] Porter TR, Xie F (2010). Myocardial perfusion imaging with contrast ultrasound. JACC Cardiovasc Imaging.

[30] Baselet B, Sonveaux P, Baatout S, Aerts A (2019). Pathological effects of ionizing radiation: endothelial activation and dysfunction. Cell Mol Life Sci.

[31] Holler V, Buard V, Gaugler MH, Guipaud O, Baudelin C, Sache A, Perez Mdel R, Squiban C, Tamarat R, Milliat F (2009). Pravastatin limits radiation-induced vascular dysfunction in the skin. J Investig Dermatol.

[32] Black MA, Green DJ, Cable NT (2008). Exercise prevents age-related decline in nitric-oxide-mediated vasodilator function in cutaneous microvessels. J Physiol.

[33] Fujii N, Reinke MC, Brunt VE, Minson CT (2013). Impaired acetylcholine-induced cutaneous vasodilation in young smokers: roles of nitric oxide and prostanoids. Am J Physiol Heart Circ Physiol.

[34] Kellogg DL, Zhao JL, Coey U, Green JV (2005). Acetylcholine-induced vasodilation is mediated by nitric oxide and prostaglandins in human skin. J Appl Physiol (Bethesda, Md : 1985).

[35] Medow MS, Glover JL, Stewart JM (2008). Nitric oxide and prostaglandin inhibition during acetylcholine-mediated cutaneous vasodilation in humans. Microcirculation (New York, NY : 1994).

[36] Hatoum OA, Otterson MF, Kopelman D, Miura H, Sukhotnik I, Larsen BT, Selle RM, Moulder JE, Gutterman DD (2006). Radiation induces endothelial dysfunction in murine intestinal arterioles via enhanced production of reactive oxygen species. Arterioscler Thromb Vasc Biol.

[37] Maynard KI, Stewart-Lee AL, Milner P, Burnstock G (1992). X-irradiation attenuates relaxant responses in the rabbit ear artery. Br J Pharmacol.

[38] Soloviev AI, Tishkin SM, Parshikov AV, Ivanova IV, Goncharov EV, Gurney AM (2003). Mechanisms of endothelial dysfunction after ionized radiation: selective impairment of the nitric oxide component of endothelium-dependent vasodilation. Br J Pharmacol.

[39] Arcangeli S, Pinzi V, Arcangeli G (2012). Epidemiology of prostate cancer and treatment remarks. World J Radiol.

[40] Michaelson MD, Cotter SE, Gargollo PC, Zietman AL, Dahl DM, Smith MR (2008). Management of complications of prostate cancer treatment. CA Cancer J Clin.

[41] Hamdy O, Abou-Elenin K, LoGerfo FW, Horton ES, Veves A (2001). Contribution of nerve-axon reflex-related vasodilation to the total skin vasodilation in diabetic patients with and without neuropathy. Diabetes Care.

[42] Rodriguez-Miguelez P, Thomas J, Seigler N, Crandall R, McKie KT, Forseen C, Harris RA (2016). Evidence of microvascular dysfunction in patients with cystic fibrosis. Am J Phys Heart Circ Phys.

[43] Walther G, Obert P, Dutheil F, Chapier R, Lesourd B, Naughton G, Courteix D, Vinet A (2015). Metabolic syndrome individuals with and without type 2 diabetes mellitus present generalized vascular dysfunction: cross-sectional study. Arterioscler Thromb Vasc Biol.

